# Biogas Production by Anaerobic Co-Digestion of Dairy Wastewater with the Crude Glycerol from Slaughterhouse Sludge Cake Transesterification

**DOI:** 10.3390/ani9090618

**Published:** 2019-08-28

**Authors:** Yu-Chun Chou, Jung-Jeng Su

**Affiliations:** 1 Department of Animal Science and Technology, National Taiwan University, Taipei 10673, Taiwan; 2 Bioenergy Research Center, College of Bioresources and Agriculture, National Taiwan University, Taipei 10617, Taiwan

**Keywords:** sludge, transesterification, biodiesel, fatty acid methyl ester (FAME), crude glycerol, anaerobic co-digestion, biogas

## Abstract

**Simple Summary:**

In our previous study, slaughterhouse sludge cake was trans-esterified to produce biofuel and some organic waste, i.e., crude glycerol. The crude glycerol was evaluated for feasibility of using crude glycerol as the feedstock to produce biofuel by anaerobic co-digestion with dairy cattle wastewater. Addition of crude glycerol significantly increased production efficiency of biogas but decreased the removal efficiency of total solids and volatile solids with increased crude glycerol volume. The results showed that the slaughterhouse sludge cake is a feasible feedstock for producing biodiesel and the waste, crude glycerol, can promote biogas production.

**Abstract:**

Excessive sludge in the wastewater treatment basins has to be removed periodically and collected as the form of sludge cake for promising good water quality of the effluent. This study aims to evaluate the feasibility of biogas production by anaerobic co-digestion of dairy cattle wastewater and crude glycerol from transesterification of sludge cake. Different ratios of crude glycerol, i.e., 2, 4, and 8% (v/v), from the previous experiment were mixed with dairy cattle wastewater and inoculated with anaerobic sludge in cap-sealed 1-L serum bottles as anaerobic digesters. Although the 8% crude glycerol set showed the highest total biogas and methane production, low pH from volatile fatty acid accumulation decreased the removal efficiency of chemical oxygen demand, biochemical oxygen demand, and suspended solids after a 14-d incubation period. The experimental sets with 2 and 4% of crude glycerol increased total methane production up to 177 and 226% compared to the control set, respectively. We found that addition of crude glycerol decreased removal efficiency of total solids and volatile solids. In our study, we proved that slaughterhouse sludge cake is a feasible feedstock for producing biogas through transesterification and anaerobic co-digestion.

## 1. Introduction

Anaerobic digestion is a microbial process to convert organics and produce methane including hydrolysis, acidogenesis, acetogenesis, and methanogenesis [1,2]. Several major factors affect the efficiency of anaerobic digestion including: temperature, pH values, volatile fatty acid (VFA), ammonium, and nitrogen to carbon ratio (C/N ratio). Different bacterial populations, such as psychrophilic, mesophilic, and thermophilic bacteria, grow under different optimal growth temperatures [3,4]. Indeed, commercial anaerobic digesters have two major operation temperature options, mesophilic and thermophilic [5,6,7]. 

The optimal pH values for anaerobic digestion, hydrolysis, and acidogenesis are about 7–8, 5.5, and 6.5, respectively [8,9]. The pH value mainly depends on the concentrations of ammonium ion and VFA. High ammonium concentration may result in increased pH value while excessive accumulation of VFA may result in decreased pH value. However, buffer substrates can optimize the pH value, such as animal manure [1]. Lots of produced VFA may result in decreased pH during acidogenesis and inhibit growth of methanogen. Conversion efficiency of VFA by the acidogenic bacteria has been reported as follows: acetic acid > ethanol > butyric acid > propionic acid [10]. Thus, propionic acid accumulates easily during methanogenesis and inhibits the growth of methanogen. When the concentrations of propionic acid reach more than 900 mg/L, the growth of methanogen and methane production is inhibited and decreased significantly [11]. Moreover, when the VFA concentration is more than 2000 and 4000 mg/L in the substrate, capability of cellulose and glucose degradation by the bacteria is inhibited, respectively [12]. Co-digestion of lipid-containing wastes with municipal wastewater sludge can greatly improve bio-methane recovery at wastewater treatment facilities. However, inhibition of anaerobic digestion using algal biomass and lipid-containing wastes such as fats, oils, and greases have been studied resulting from the inhibitory effects of long chain fatty acid (LCFA) (palmitic, stearic, and oleic acid) accumulation [13,14,15]. 

During the anaerobic digestion (AD) process, lipids are initially hydrolyzed to LCFAs and glycerol in a fast step by extracellular lipases excreted by hydrolytic bacteria. LCFAs then adsorb to and are transported within microbial cell membranes. Once inside, LCFAs are further degraded to acetic acid and hydrogen through β-oxidation by syntrophic acetogenic bacteria. The difference between the rates of hydrolysis of lipids and β-oxidation of LCFAs could result in a reactant-product imbalance and LCFA accumulation over time, resulting in inhibition on microbial activity [14]. 

The inhibitory effect of LCFAs on microbial activity of hydrolytic bacteria, acidogens, acetogens, and methanogens within anaerobic consortium has been well documented. Methanogens were reported to be more susceptible to LCFA inhibition compared to acidogens, while acetotrophic methanogens are reported to be more severely affected than hydrogenotrophic methanogens. If the microbial population is disrupted by LCFAs, inhibited digestion will occur, leading to VFA accumulation and depressed methane production [14].

A previous study found that increased temperature improved the methane potential, but the rate was reduced from mesophilic to thermophilic conditions, due to the accumulation of ammonium nitrogen and free ammonia and the occurrence of ammonia inhibition [16]. When temperature increased, an increase was required in the feed C/N ratio, in order to reduce the risk of ammonia inhibition. The increase of C/N ratios reduced the negative effects of ammonia and maximum methane potentials were achieved with C/N ratios of 25 and 30 at 35 °C and 55 °C, respectively [16].

Co-digestion is a waste treatment method in which different wastes are mixed and treated together. The benefits of co-digestion include: dilution of potential toxic compounds, improved balance of nutrients, synergistic effect of anaerobes, increased load of biodegradable organics and better biogas production [8,17].

Crude glycerol is a by-product from biodiesel industry. For every 9 kg of biodiesel produced, about 1 kg of a crude glycerol by-product is formed [18]. Crude glycerol generated by homogeneous base-catalyzed transesterification contains approximately 50–60% of glycerol, 12–16% of alkalis, especially in the form of alkali soaps and hydroxides, 15–18% of methyl esters, 8–12% of methanol, 2–3% of water and further components [19]. There are several possible conversions of crude glycerol into useful products such as 1,3-propanediol, 1,2-propanediol, dihydroxyacetones, hydrogen, polyglycerols, succinic acid, and polyesters. Crude glycerol, when used in combination with other compounds, yields other useful products [20].

The typical elemental analysis of crude glycerol generated by biodiesel industry indicated that the major elemental contents of this material are C (52.77%), H (11.08%), and O (36.15%) [21]. The feasibility of adding crude glycerol from the biodiesel industry to the anaerobic digesters treating sewage sludge in wastewater treatment plants was studied. The reactor treating the sewage sludge produced 1106 ± 36 mL CH_4_/d before the addition of glycerol and 2353 ± 94 mL CH_4_/d after the addition of glycerol (1% v/v in the feed). The results suggested that the extra addition of glycerol in the feedstock (about 8% v/v) resulted in an accumulation of propionate in the reactors and, finally, in the instability of the system [22].

Anaerobic digestion of crude glycerol with dairy slurry was carried out in four laboratory-scale CSTR-type biogas digesters with a working volume of 3 L at 35–37 °C for a 10-week period. Compared to the control, methane contents were increased by 9.5, 14.3, and 14.6%, respectively, for the treatments of 5, 10, and 15% crude glycerol addition [23]. 

Biogas production by co-digestion of cattle manure and crude glycerol was studied after pre-treatment of the cattle manure or mixtures of cattle manure with different amounts of added glycerol with ultrasound. Under mesophilic conditions, the addition of 4% glycerin to screened cattle manure increased biogas production by up to 400%. Application of sonication (20 kHz, 0.1 kW, and 4 min) to a mixture of manure plus 4% glycerol increased biogas production by up to 800% compared to untreated manure. The best results were obtained under thermophilic conditions using sonicated mixtures of ground cattle manure with 6% added glycerol [24]. 

Thus, the objective of this study was to establish the optimal parameters for co-digestion of crude glycerol from trans-esterification of sludge cake with dairy cattle wastewater for recycling crude glycerol to produce biogas.

## 2. Material and Methods

### 2.1. Crude Glycerol Collection and Preparation

To summarize the results of our previous study, the sludge cake was collected from a selected commercial slaughterhouse and trans-esterified with methanol, n-hexane, and acidic catalysts at 55 °C. Results showed that the highest fatty acid methyl ester (FAME) yield achieved when 4% (v/v) of sulfuric acid or hydrochloric acid were added for a 24-h reaction, individually. Methyl esters of palmitic acid (C16:0), palmitoleic acid (C16:1), stearic acid (C18:0), and oleic acid (C18:1) were the major components of biodiesel from transesterification of slaughterhouse sludge cake. However, certain amounts of palmitic acid, palmitoleic acid, stearic acid, and oleic acid might remain in the crude glycerol and cause inhibitory effects of anaerobic digestion [15]. The n-hexane was removed from crude glycerol after centrifugation of crude glycerol layer at 4000 rpm for 6 min. Before anaerobic digestion of crude glycerol, the crude glycerol was neutralized by adding calcium hydroxide solution and the sediment (calcium sulfate) was filtrated after centrifuged at 4000 rpm for 15 min. The filtrate of crude glycerol was then stored at room temperature for further studies. The dairy cattle wastewater after solid/liquid separation and anaerobic sludge from the digesters of dairy wastewater treatment facility were taken from the livestock farm of National Taiwan University (NTU) and used as the primary feedstock and inocula for anaerobic digestion, respectively.

### 2.2. Design of Digesters for Co-Digestion of Crude Glycerol and Dairy Cattle Wastewater

**Figure 1:**
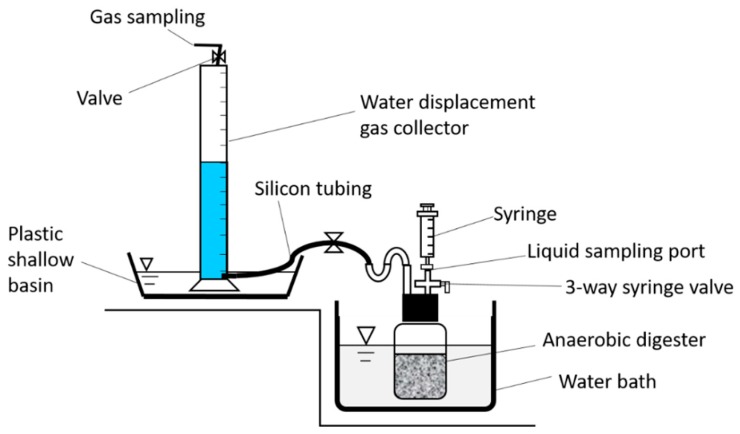
Sketch of the digester design.

Gas-tight screw-cap serum bottles (1 L serum bottle) with an *S*-shaped glass tubing connecting to a water displacement gas collector (water pH = 2.0) were used as anaerobic digester in this study. All gas-tight serum bottles with feedstock were incubated in a water bath at 35 °C during the experimental periods (Figure 1). There was a three-way syringe valve with Luer lock on the top of the screw cap of all serum bottles for gas sampling periodically. The syringe valve was fixed on the top of the screw cap by filling with silica gel to prevent leaking from the capped serum bottles. After filling with a mixture of anaerobic sludge, dairy wastewater, and crude glycerol, oxygen can be used up by facultatively anaerobic bacteria inside the gas-tight serum bottles in 24 h. 

### 2.3. Time Course Experiments of Co-Digestion using Crude Glycerol and Dairy Cattle Wastewater as Feedstock

The feedstock for co-digestion was a mixture of dairy sludge and dairy cattle wastewater with different ratios of crude glycerol addition (Table 1). The control set was a mixture of 300 mL dairy sludge and 620–680 mL of dairy cattle wastewater without adding any crude glycerol. For the addition ratios of 2, 4, and 8% (v/v) sets, there was 20, 40, and 80 mL of crude glycerol added into dairy cattle wastewater mixture, respectively. They were assigned as CG2, CG4, and CG8 for this study, respectively. The time course experiments were performed in triplicates in a 14-d period and the liquid samples were taken at the Days 0 and 14 for determination of chemical oxygen demand (COD), biochemical oxygen demand (BOD), suspended solids (SS), total solids (TS), volatile solids (VS), pH, and Volatile Fatty Acids (VFAs). Biogas production was recorded daily and gas samples were taken periodically and analyzed by gas chromatography. 

### 2.4. Sample Analysis 

#### 2.4.1. Water Quality of Wastewater Samples

Wastewater samples were analyzed for COD, BOD, SS, TS, and VS using Standard Methods [25]. The pH of the samples was determined by a pH meter (PH200, CLEAN instruments Co., Ltd., New Taipei City, Taiwan). The values of VS are in dry basis.

#### 2.4.2. Gas Contents of Gas Samples

Biogas samples were analyzed for their composition by gas chromatography (Master GC, DANI Instruments, Marlborough, MA, USA), which was equipped with a thermal conductivity detector (TCD) and Carboxen 1010 PLOT capillary column (30 m × 0.53 mm × 0.25 μm film thickness; Supelco Analytical of Sigma-Aldrich Co., Bellefonte, PA, USA) [26]. The sample injection volume was 250 μL and was injected using Pressure-Lok^®^ analytical syringes (VICI, Valco Instruments Co., Ltd., Houston, TX, USA). 

#### 2.4.3. Volatile Fatty Acids of Liquid Samples

The liquid sample (2 mL) was mixed with 500 μL of 20% H_3_PO_4_. The mixture was next centrifuged (Z 36HK, Hermle Labortechnik GmbH, Germany; radius of rotor = 10 cm) for 20 min at 4 °C with 15,000 rcf to remove solids in the solution. The supernatant was filtered through a 0.2-μm filter and transferred to a 2 mL gas chromatography vial. The identification of the volatile fatty acids (VFAs) of the effluent was conducted using gas chromatography (Agilent GC 7820A, Agilent Technologies) equipped with a flame ionization detector (FID) and a Nukol capillary column (30 m × 0.25 mm × 0.25 μm film thickness, Supelco) [25]. The WSFA-2 Mix (Sigma-Aldrich) was applied as the standard for calibrating acetic acid, propionic acid, butyric acid, isobutyric acid, valeric acid, and isovaleric acid.

### 2.5. Statistical Analysis

The experimental data of different samples were then analyzed using the ANOVA procedure of data analyzing and graphing software, Prism 6 (GraphPad Software, San Diego, CA, USA) and Origin (OriginLab, Northampton, MA, USA), respectively.

## 3. Results and Discussion

### 3.1. Analysis of Feedstock for Anaerobic Co-Digestion

The feedstock for this study was dairy wastewater and crude glycerol. Dairy sludge was used as the inocula for anaerobic digestion. The pH, moisture, TS, and VS for all feedstock and experimental sets are shown in Table 2. The TS and VS for all sets were 1.52–3.15% and 63.24–77.85%, respectively. The feedstock of the 2, 4, and 8% crude glycerol addition was prepared by deionized water and crude glycerol from transesterification of slaughterhouse sludge cake. The pH of all crude glycerol addition sets was 7.07–7.56 depending on addition ratio of acidic crude glycerol. The more crude glycerol added, the lower the pH of the feedstock retained. The acidic crude glycerol was obtained from acidic catalyzed transesterification of sludge cake. Applying certain amounts of Ca(OH)_2_ on acidic crude glycerol can help to eliminate SO_4_^2−^ by chemical precipitation.

### 3.2. Biogas Production During the Co-Digestion of Dairy Cattle Wastewater and Crude Glycerol

**Figure 2:**
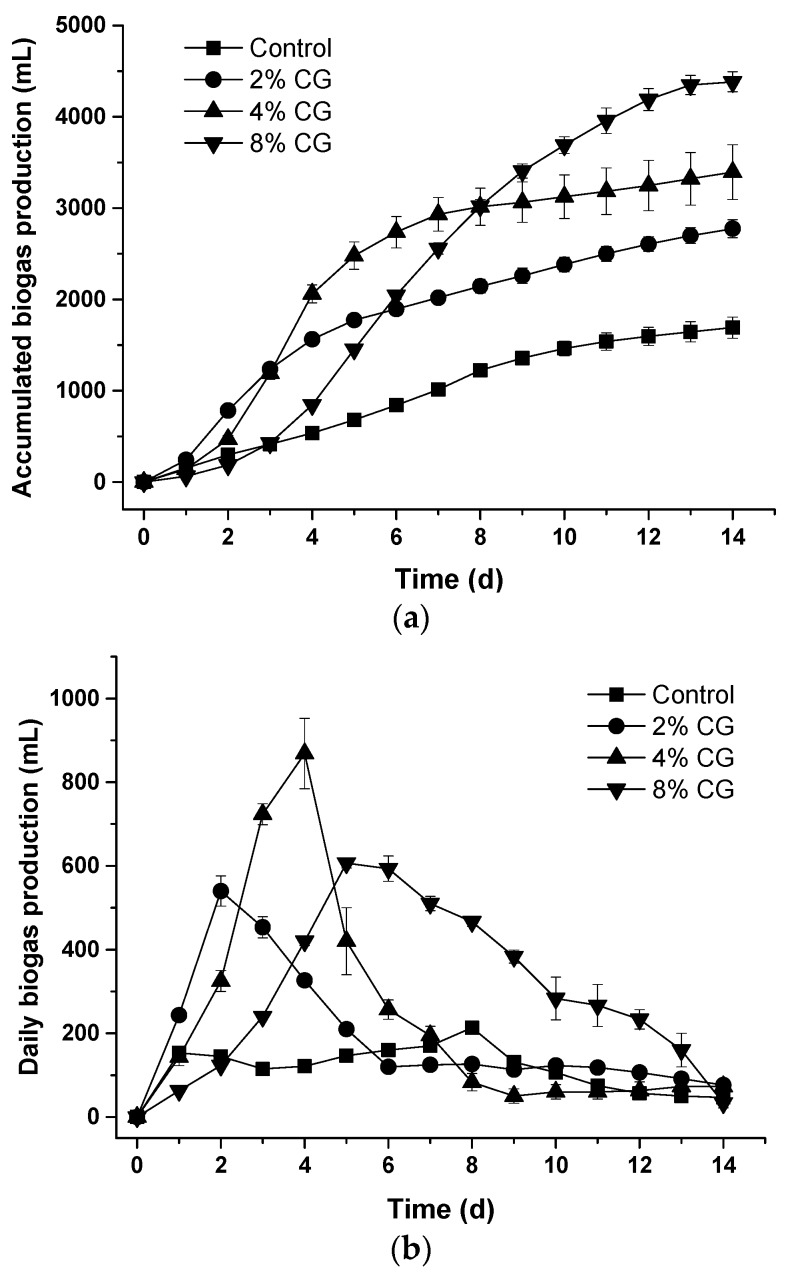
Effects of adding different percentage of crude glycerol on (**a**) daily biogas production and (**b**) accumulated biogas production.

The daily biogas production from the control set was much lower than the other treatment sets. The highest peak of daily biogas production for the CG2, CG4, and CG8 sets was at Day 2, Day 4, and Day 5, respectively (*p* < 0.05) (Figure 2a). However, the total accumulated biogas production for the CG2, CG4, and CG8 sets was 2775.0 ± 97.3, 3395.0 ± 300.5, and 4383.3 ± 110.2 mL, respectively, compared to the control set 1691.7 ± 115.6 mL (*p* < 0.05) (Figure 2b). The more crude glycerol added, the more the total accumulated biogas production obtained. All biogas and methane production resulted from acetic acid utilization by acetogenic bacteria in the control and all crude glycerol addition sets (Figure 6).

**Figure 3:**
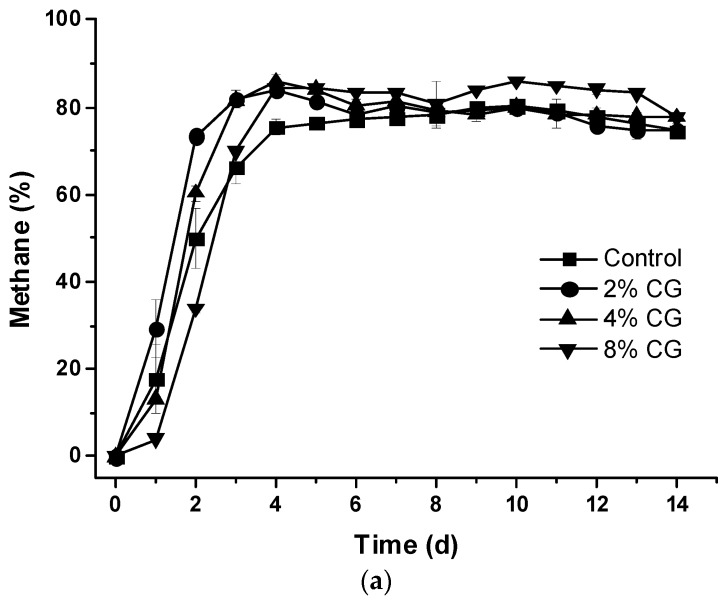

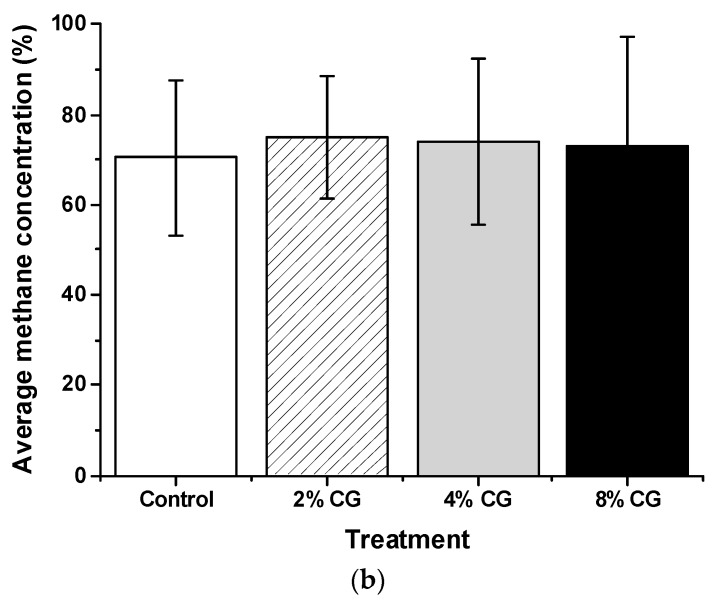
Effects of adding different percentage of crude glycerol on (**a**) daily methane concentration and (**b**) average methane concentration.

The methane concentrations increased rapidly in 4 d and were all higher than 70% in the biogas for all sets of anaerobic co-digestion in the 14-d period (*p* > 0.05) (Figure 3a). Moreover, the average methane concentrations were 74.99 ± 13.52%, 73.97 ± 18.44%, and 73.10 ± 24.03%, respectively, compared to the control set 70.40 ± 17.06% with no significant difference (*p* > 0.05) (Figure 3b). 

**Figure 4:**
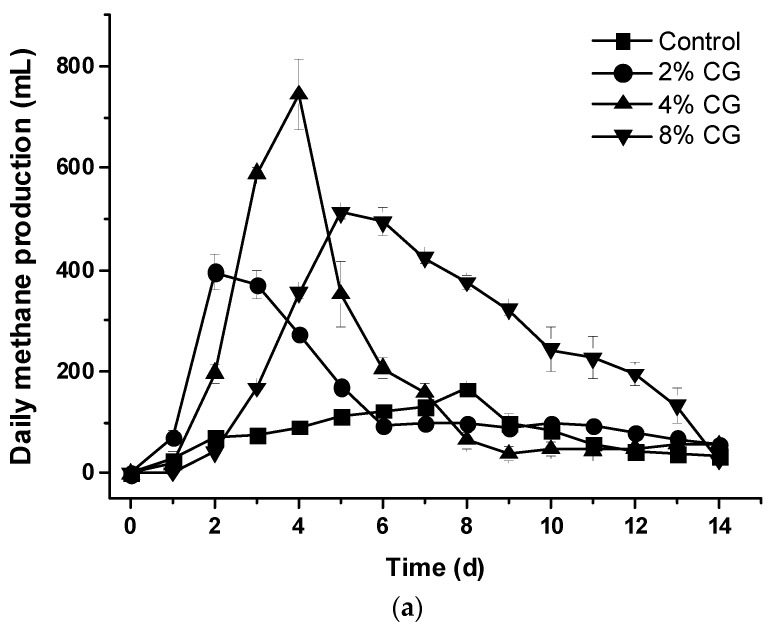

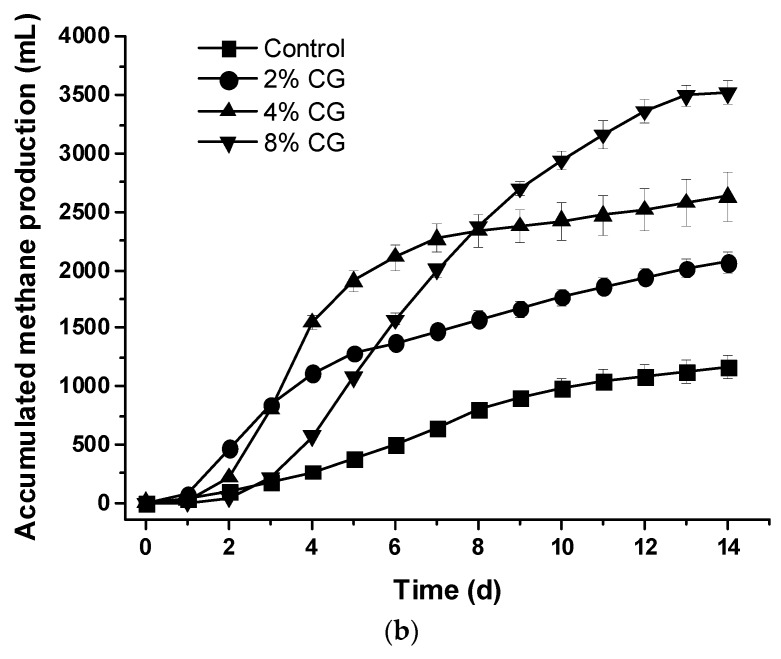
Effects of adding different percentage of crude glycerol on (**a**) daily methane production and (**b**) accumulated methane production.

The daily methane production from the control set was much lower than the other treatment sets. The highest peak of daily methane production for the CG2, CG4, and CG8 sets was at Day 2, Day 4, and Day 5, respectively (*p* < 0.05) (Figure 4a). However, the total accumulated methane production for the CG2, CG4, and CG8 sets was 2064.0 ± 85.3, 2629.6 ± 209.4, and 3520.5 ± 95.3 mL, respectively, compared to the control set 1162.9 ± 102.4 mL (*p* < 0.05) (Figure 4b). The more crude glycerol added, the more the total accumulated methane production obtained.

**Figure 5:**
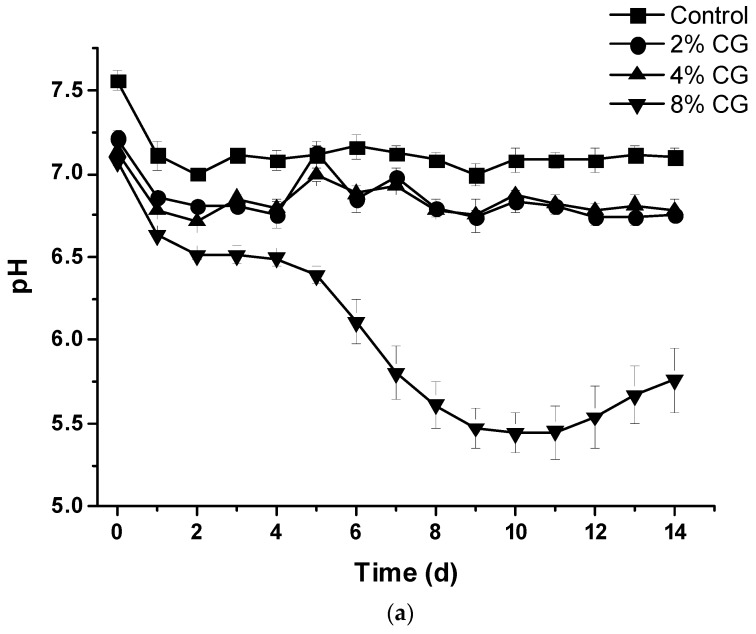

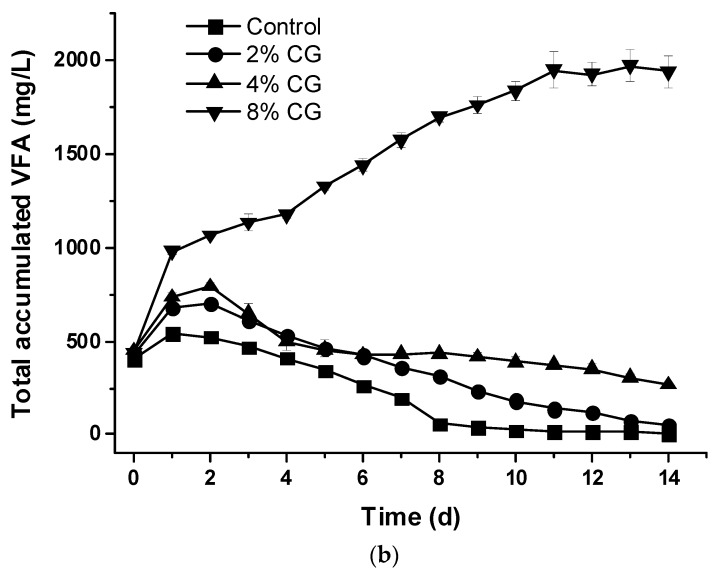
(**a**) Daily pH value and (**b**) accumulated total volatile fatty acid of adding different percentage of crude glycerol.

**Figure 6:**
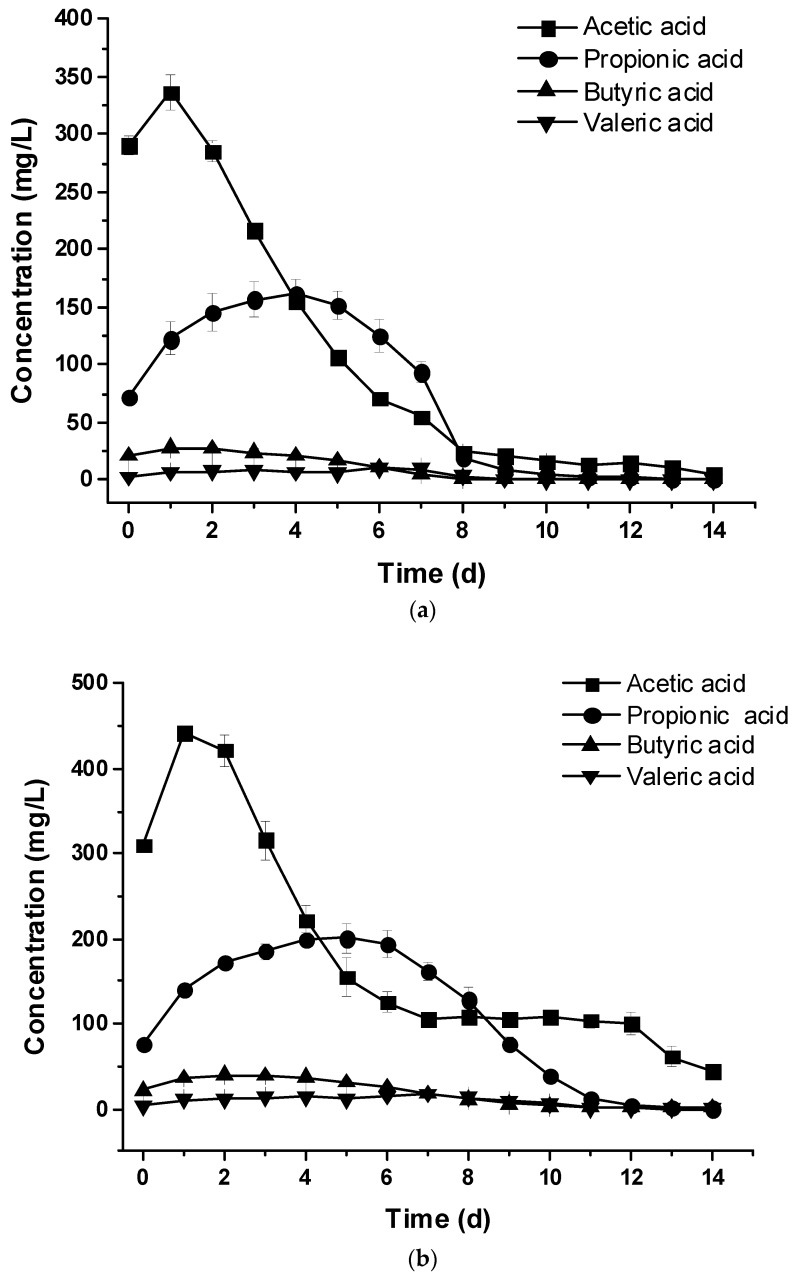

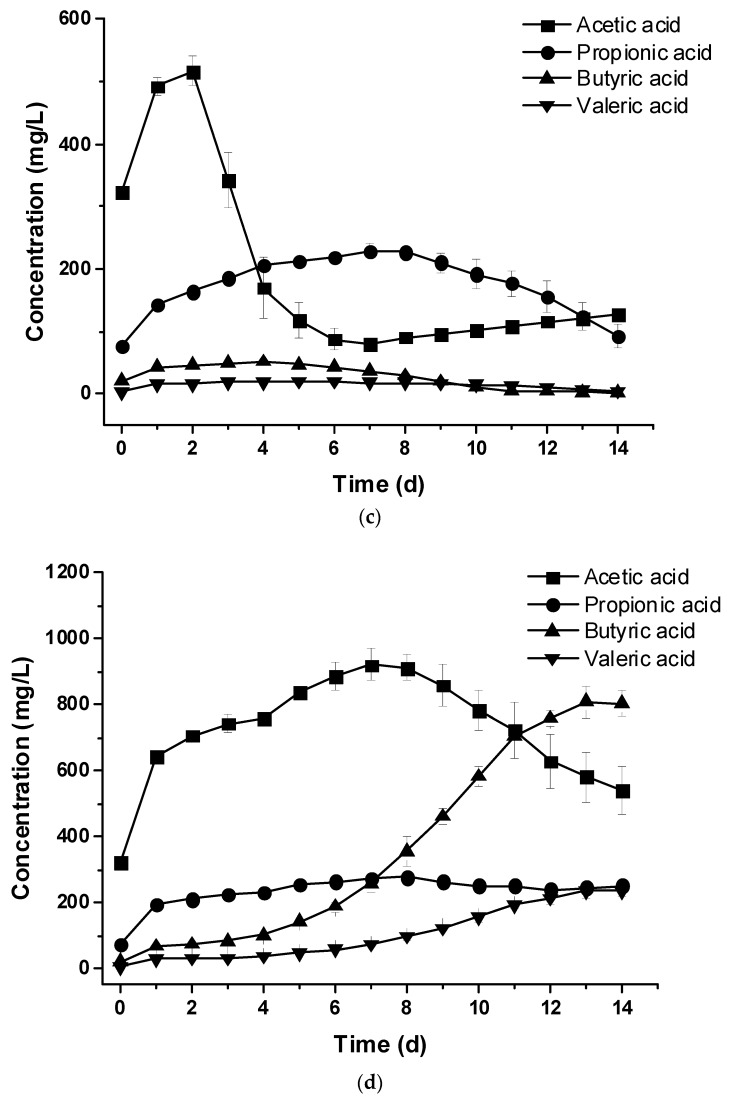
Changes of accumulated volatile fatty acid without (**a**), with 2% (**b**), 4% (**c**), and 8% (**d**) crude glycerol addition.

Except the CG8 set, the pH of all other sets was above 6.7. The pH of the CG8 set decreased rapidly after 5 d from pH = 7.07 to lower than 6.0 (Figure 5a) and the final pH of the feedstock was 5.76 ± 0.19 in the 14-d period. Results showed that it resulted from quick accumulation of the VFA, butyric acid, in the digesters during anaerobic digestion (Figure 5a,b and Figure 6d). The amount of VFA increased rapidly after the Day 2, however, the amount of VFA decreased for all other treatment sets (Figure 5b). The results implied that the daily biogas production decreased resulting from the accumulation of VFA and low pH of the feedstock after Day 6 in a 14-d period (Figure 2). The optimal pH for anaerobic digestion was studied and defined as 6.8–7.2 [1].

Due to hydrolysis and acidogenesis, and acetogenesis of the feedstock, the total amount of accumulated VFA increased in 3 d and then decreased resulting from proliferation of methanogen after 4 d for the control, CG2, and CG4 sets in the 14-d period (Figure 5b). For the CG8 set, the pH of the feedstock decreased dramatically after 4 d due to rapid accumulation of VFA in the feedstock. This might imply that the low pH inhibited the growth of methanogen in the digester resulting in VFA accumulation. 

The study of Siegert and Banks [12] showed that VFA caused inhibition of the cellulolytic activity at concentrations <2000 mg/L, and therefore, of the rate of cellulose hydrolysis. Moreover, the fermentation of glucose was slightly inhibited at VFA concentrations above 4000 mg/L. 

For the CG8 set, total accumulated VFA was 1179.68 ± 20.06 mg/L at Day 5. However, total accumulated VFA rapidly increased from 1331.11 ± 24.45 to 1973.01 ± 89.40 mg/L (from Day 6 to Day 14) (Figure 5b). The amount of VFA in the feedstock was retained to the inhibitory limit (about 2000 mg/L). Daily biogas as well as methane production was also inhibited and coordinated with rapid accumulation of VFA from Day 6 (Figure 2a, Figure 4a and Figure 5b). 

At the highest concentrations of acetic acid and butyric acid, 2400 and 1800 mg/L respectively, no significant inhibition of the activity of methanogenic bacteria was observed. However, when the propionic acid concentration was increased to 900 mg/L, significant inhibition of the bacterial growth was observed. The effect resulted in the accumulation of VFAs, and the total methane production became very low [11]. From the analyzed results of the VFA samples, acetic acid was the first accumulated VFA component compared to other components including propionic acid, butyric acid, and valeric acid (Figure 6a–d). The propionic acid and butyric acid are transformed by acetogenic bacteria to H_2_, CO_2_, and acetic acid.

For the control set without adding crude glycerol, the highest peak of acetic acid and propionic acid was 336.57 ± 15.07 and 161.32 ± 12.02 mg/L at the Days 1 and 4, respectively (Figure 6a). For the CG2 and CG4 sets, the highest peak of acetic acid was 442.65 ± 6.20 and 516.09 ± 23.53 mg/L at Days 1 and 2, respectively (Figure 6b–c). The highest peak of propionic acid was 200.46 ± 18.51 and 229.34 ± 11.8 mg/L at Days 5 and 7, respectively (Figure 6b–c). For the CG8 set, the highest peak of acetic acid, propionic acid, and butyric acid was 920.34 ± 148.90, 276.90 ± 12.48, and 808.6 ± 48.7 mg/L at Days 7, 8, and 13, respectively (Figure 6d). The highest concentrations of acetic acid and butyric acid, and propionic acid were less than the inhibitory limits (i.e., acetic acid < 2400, butyric acid < 1800, and propionic acid < 900 mg/L) [11]. As a result, accumulation of VFA might not be the key factor to inhibit the daily biogas and methane production. The decreased pH values result from increased butyric acid and lack of buffer capability in the feedstock of the CG8 digesters.

### 3.3. Removal Efficiency of TS and VS During the Co-Digestion Process

**Figure 7:**
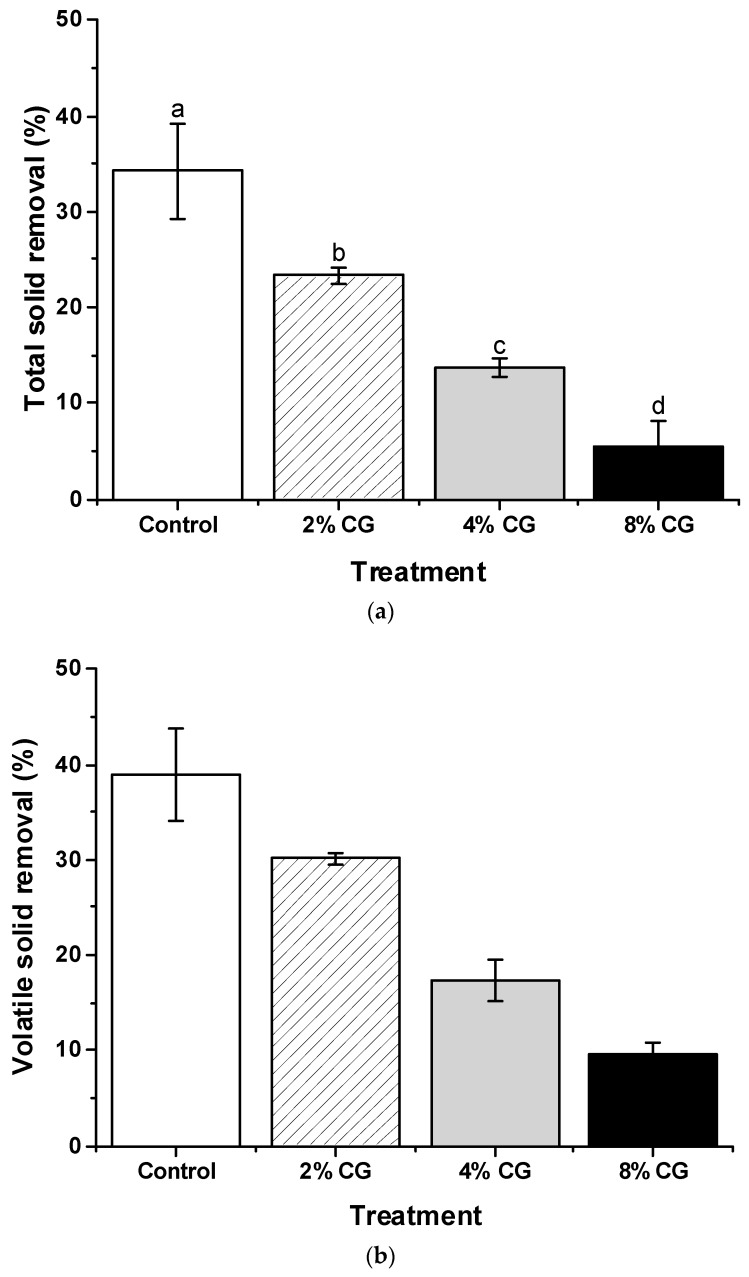
Changes of total solids (TS) (**a**) and volatile solids (VS) (**b**) without or with different percentage of crude glycerol addition.

Experimental results showed that the TS removal efficiency of the control, CG2, CG4, and CG8 sets were 38.9 ± 4.9%, 30.2 ± 0.6%, 17.4 ± 2.2%, and 9.5 ± 1.4% in the 14-d period of anaerobic digestion, respectively (Figure 7a). The more crude glycerol added, the lower the TS removal efficiency obtained (*p* < 0.05). Part of the VS, acetic acid, can be utilized by acidogenic and acetogenic bacteria to produce methane through anaerobic digestion [1]. Experimental results showed that VS removal efficiencies of the control, CG2, CG4, and CG8 sets were 34.2 ± 4.9%, 23.3 ± 0.9%, 13.7 ± 1.0%, and 5.5 ± 2.7% in the 14-d period of anaerobic digestion, respectively (Figure 7b). A similar phenomenon was observed which is the more crude glycerol added, the lower the VS removal efficiency obtained (*p* < 0.05). In brief summary, addition of crude glycerol decreased the removal efficiency of TS and VS but increased biogas production. This might indicate that the heterotrophic bacteria in the digesters prefer the readily degraded VFAs rather than either VS or TS to maintain their metabolism. In addition, some organic matters of the TS or VS might not be biodegradable.

### 3.4. Comparison of the Specific Biogas Production Among the CG2, CG4, and CG8 Sets During the Co-Digestion Process

**Figure 8:**
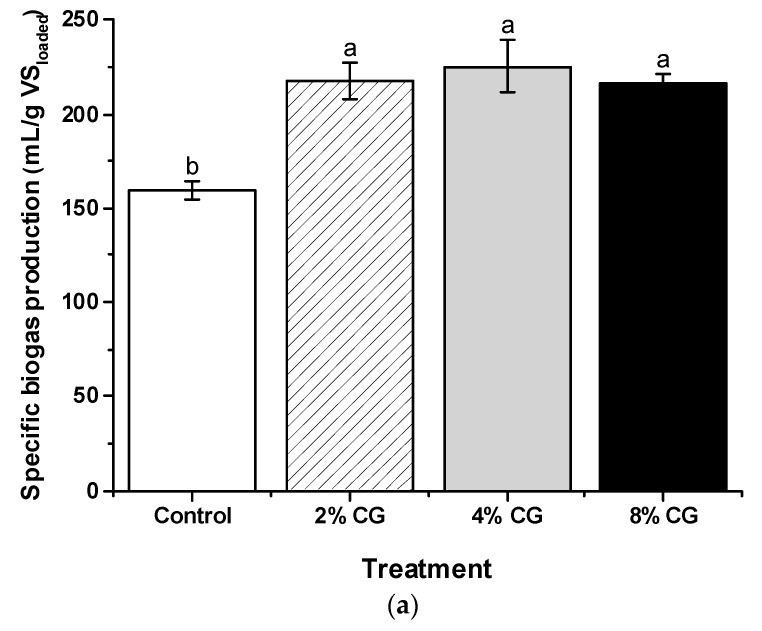

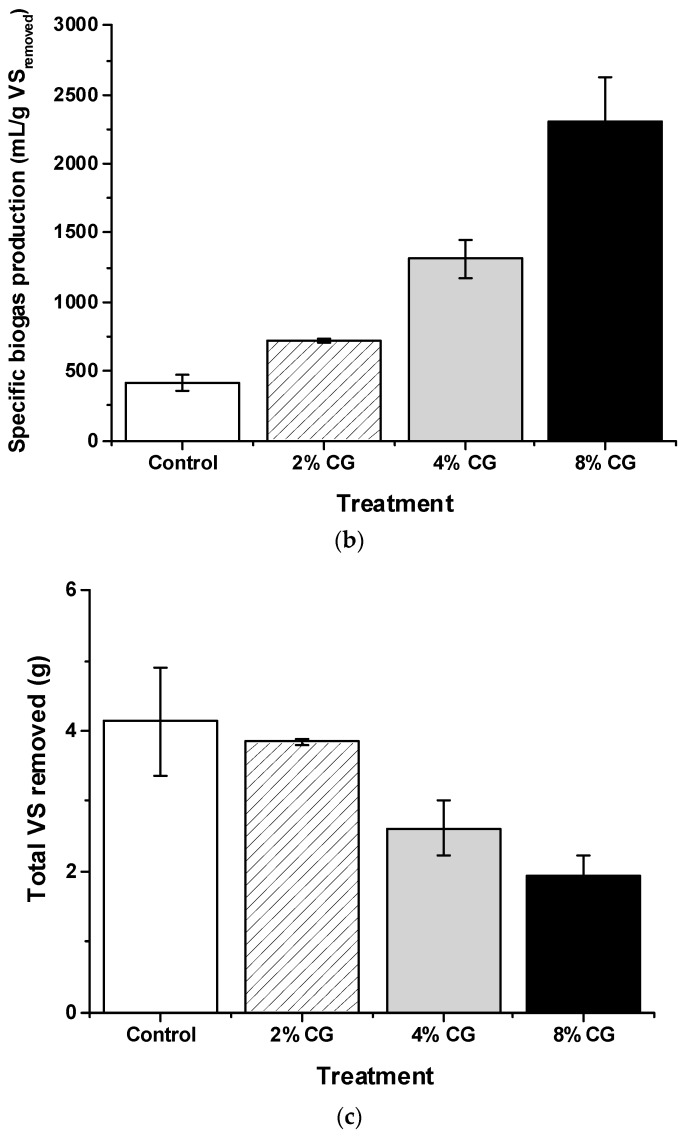
Changes of biogas production rates depends on VS loaded (**a**), VS removed (**b**), and total amount of VS removed (**c**) without or with different percentage of crude glycerol addition.

We define the specific biogas production based one two parameters, the total initially loaded VS (VS_loaded_) and total removed VS (VS_removed_) during the anaerobic digestion. The specific biogas production increased with the addition of crude glycerol based on the data of the VS_loaded_ for the control, CG2, CG4, and CG8 was 159.4 ± 5.2, 217.8 ± 9.1, 225.6 ± 13.7, and 216.0 ± 5.2 mL/g-VS_load_, respectively. The more crude glycerol added, the higher specific biogas production obtained (*p* < 0.05) (Figure 8a). There was significant difference between the control and all crude glycerol addition sets (*p* < 0.05), but no significant difference on the specific biogas production among the CG2, CG4, and CG8 sets (*p* > 0.05) (Figure 8a). 

Furthermore, results showed that the decreased total VS amount removed during the 14-d period of anaerobic digestion comes with the increased addition amount of crude glycerol addition (Figure 8c). The specific biogas production increased with the addition of crude glycerol based on the data of the VS_removed_ for the control, CG2, CG4, and CG8 was 414.9 ± 57.0, 722.0 ± 20.6, 1310.0 ± 144.4, and 2307.2 ± 312.8 mL/g-VS_removed_, respectively. Thus, the increased specific biogas production comes with the decreased total VS amount removed during the 14-d period of anaerobic digestion. It implies that the more crude glycerol added, the higher specific biogas production obtained (*p* < 0.05) (Figure 8b). In addition, there was significant difference on the specific biogas production among the CG2, CG4, and CG8 sets (*p* < 0.05) but no significant difference between the control and CG2 sets (*p* > 0.05) (Figure 8b). If the microbial population is disrupted by LCFAs, inhibited digestion will occur, leading to volatile fatty acids (VFA) accumulation and depressed methane production [14]. A certain amount of LCFAs in the crude glycerol might lead to VFA accumulation and inhibitory biogas production. Results showed that the CG8 set added more crude glycerol than the other sets (CG2 and CG4) resulting in the lowest VS removal efficiency (Figure 8c). Thus, the CG8 set achieved the highest specific biogas production based on the amount of VS removal (Figure 8b).

### 3.5. Changes of Water Quality Indexes During Anaerobic Co-Digestion of Crude Glycerol and Cattle Wastewater

Experimental results showed that the final COD concentration in the liquid digestate of all digesters was 614.4 ± 93.3, 3057.8 ± 304.2, 6304.2 ± 598.3, and 14,316.0 ± 189.1 mg/L for the control, CG2, CG4, and CG8 sets, respectively (Table 3). Thus, the COD removal efficiency for all digesters was 37.9 ± 8.9%, 59.8 ± 5.3%, 52.0 ± 3.2%, and 36.9 ± 1.9% during the 14-d period of anaerobic digestion, respectively. Addition of crude glycerol significantly increased the concentrations of COD in the digesters of all sets (*p* < 0.05) (Table 3). Compared to the COD removal efficiency of the control set with the CG2 and CG4 sets, addition of crude glycerol significantly promoted COD removal efficiency except the CG8 set (*p* < 0.05). 

Experimental results showed that the final BOD concentration in the liquid digestate of all digesters was 108.0 ± 19.4, 309.5 ± 34.7, 559.4 ± 31.9, and 3830.3 ± 176.7 mg/L for the control, CG2, CG4, and CG8 sets, respectively (Table 3). Thus, the BOD removal efficiency for all digesters was 91.2 ± 1.3%, 90.9 ± 0.8%, 90.6 ± 0.7%, and 61.9 ± 1.5% during the 14-d period of anaerobic digestion, respectively. Addition of crude glycerol significantly increased the concentrations of BOD in the digesters of all sets (*p* < 0.05) (Table 3). Compared to the BOD removal efficiency of the control set with the CG2 and CG4 sets, addition of crude glycerol did not significantly promote BOD removal efficiency (*p* > 0.05). The BOD removal efficiency of the CG8 set was the worst among all crude glycerol addition sets. 

Experimental results showed that the final SS concentration in the liquid digestate of all digesters was 66.7 ± 23.1, 100.0 ± 13.2, 156.7 ± 27.5, and 610.0 ± 10.0 mg/L for the control, CG2, CG4, and CG8 sets, respectively (Table 3). Thus, the SS removal efficiency for all digesters was 75.6 ± 7.2%, 76.4 ± 4.5%, 68.4 ± 1.6%, and 12.0 ± 1.8% during the 14-d period of anaerobic digestion, respectively. Addition of crude glycerol increased the concentrations of SS in the digesters of all sets (*p* < 0.05) (Table 3). Compared to the SS removal efficiency of the control set with the CG2 and CG4 sets in all digesters, addition of crude glycerol did not significantly promote SS removal efficiency (*p* > 0.05). Indeed, the SS removal efficiency of the CG8 set was the worst among all crude glycerol addition sets.

In a brief summary, addition of crude glycerol significantly increased the concentrations of COD, BOD, and SS in all experimental sets (*p* < 0.05) (Table 3). There was no significant difference on BOD and SS removal efficiency of the control, CG2, and CG4 sets. However, there was significant difference on COD removal efficiency among the control, CG2, and CG4 sets. The effluents from the digesters of the control, CG2, CG4, and CG8 sets need to be polished by activated sludge system to become qualified effluents. 

The study of Fountoulakis et al. [22] applied crude glycerol, the by-product from biodiesel production, with dairy cattle wastewater for co-digestion experiments under mesophilic conditions. Their experimental results showed that addition of 1% (v/v) crude glycerol increased methane production by 112%. By comparing our results with those of Fountoulakis’s study, we found that addition of 2% and 4% crude glycerol (i.e., CG2 and CG4) increased methane production by 117% and 226%, respectively [22].

The study of Castrillón et al. [24] applied sonicated mixture of cattle manure and crude glycerol from biodiesel plant as the feedstock for co-digestion experiments under mesophilic conditions. Their experimental results showed that the specific biogas production was 235.1, 168.9, and 96.7 mL/g VS for the sets with addition of 2, 4, and 8% crude glycerol, respectively [24]. By comparing our results with those of Castrillón’s study, we found that the specific biogas production in our study was 217.8 ± 9.1 and 225.6 ± 13.7 mL/g VS for the addition of 2% and 4% crude glycerol (i.e., CG2 and CG4), respectively [24]. However, the average methane content in the biogas of the CG2, CG4, and CG8 sets was 57.8%, 58.7%, and 58.4% [24] compared with 75.0 ± 13.5%, 74.0 ± 18.4%, and 73.1 ± 24.0% of our study, respectively. Thus, the results of our study show higher methane content compared to Castrillón’s study [24] and without sonication of the feedstock.

## 4. Conclusions

In this study it was proved that crude glycerol from biodiesel production process could be recycled as a feedstock to promote methane production during the co-digestion process with dairy cattle wastewater. Experimental results showed that appropriate addition of recycled crude glycerol, from transesterification of slaughterhouse sludge cake, in the digesters for co-digestion can increase biogas production and methane concentration. The best crude glycerol addition ratio to the dairy cattle wastewater for digesters was 4% (v/v) based on the high COD, BOD, and SS removal efficiency in coordination with high methane concentration in the biogas and specific biogas production. The low pH of the feedstock resulting from inhibitory effect of LCFAs on microbial activity of hydrolytic bacteria, acidogens, acetogens, and methanogens as well as accumulation of butyric acid did affect the removal efficiency of the COD, BOD, SS, TS, and VS in the CG8 digesters. The achievement of this study of converting the waste crude glycerol into renewable biogas production can be applied in the near future to the slaughterhouse industry as an advanced approach of renewable application of slaughterhouse sludge cake.

## Figures and Tables

**Table 1:** Contents of the feedstock in digesters for anaerobic co-digestion.

Feedstock (mL)	Control Set	Experimental Sets		
		CG2	CG4	CG8
Cattle Sludge (as inoculum)	300	300	300	300
Dairy cattle wastewater	700	680	660	620
Crude glycerol	0	20	40	80
Total (mL)	1000	1000	1000	1000

**Table 2:** Characteristics of feedstock for anaerobic co-digestion.

Parameters	Sludge	Dairy Wastewater	Crude Glycerol	Control	CG2	CG4	CG8
pH	7.16	7.86	6.83	7.56 ± 0.06	7.21 ± 0.05	7.13 ± 0.04	7.07 ± 0.03
Moisture (%)	96.91	99.45	75.56	98.48 ± 0.08	98.06 ± 0.05	97.63 ± 0.01	96.85 ± 0.07
TS (%)	3.09	0.55	24.44	1.52 ± 0.08	1.94 ± 0.05	2.37 ± 0.01	3.15 ± 0.07
VS * (%)	80.71	77.84	54.42	77.85 ± 0.27	71.46 ± 0.46	67.93 ± 0.71	63.24 ± 1.14

^*^ The values of VS are in dry basis. TS: total solids. VS: volatile solids.

**Table 3:** Changes of the chemical oxygen demand (COD), biochemical oxygen demand (BOD), and suspended solids (SS) removal efficiency for the liquid digestate of all digesters with different addition ratio of crude glycerol.

Experimental Sets	Day 0	Day 14	Removal (%)
	COD (mg/L)		
Control	2598.7 ± 212.5 ^d^	1614.4 ± 93.3 ^d^	37.9 ± 8.9 ^b^
2% CG	7598.9 ± 362.7 ^c^	3057.8 ± 304.2 ^c^	59.8 ± 5.3 ^a^
4% CG	13134.2 ± 370.1 ^b^	6304.2 ± 598.3 ^b^	52.0 ± 3.2 ^a^
8% CG	22689.0 ± 399.7 ^a^	14316.0 ± 189.1 ^a^	36.9 ± 1.9 ^b^
*p*-value	<0.05	<0.05	NS
	**BOD (mg/L)**		
Control	1219.1 ± 53.5 ^d^	108.0 ± 19.4 ^d^	91.2 ± 1.3 ^a^
2% CG	3417.5 ± 190.5 ^c^	309.5 ± 34.7 ^c^	90.9 ± 0.8 ^a^
4% CG	5954.0 ± 129.5 ^b^	559.4 ± 31.9 ^b^	90.6 ± 0.7 ^a^
8% CG	10065.0 ± 420.8 ^a^	3830.3 ± 176.7 ^a^	61.9 ± 1.5 ^b^
*p*-value	<0.05	<0.05	NS
	**SS (mg/L)**		
Control	270.0 ± 36.1 ^c^	66.7 ± 23.1 ^b^	75.6 ± 7.2 ^a^
2% CG	426.7 ± 23.1 ^b^	100.0 ± 13.2 ^b^	76.4 ± 4.5 ^a^
4% CG	493.3 ± 64.3 ^b^	156.7 ± 27.5 ^b^	68.4 ± 1.6 ^a^
8% CG	693.3 ± 23.1 ^a^	610.0 ± 10.0 ^a^	12.0 ± 1.8 ^b^
*p*-value	NS	NS	NS

Different letters in the same column indicate statistical significance at 5% level at Tukey test. NS: not significant.
